# Pharmacodynamic and Metabolomics Studies on the Effect of Kouyanqing Granule in the Treatment of Phenol-Induced Oral Ulcer Worsened by Sleep Deprivation

**DOI:** 10.3389/fphar.2020.00824

**Published:** 2020-06-30

**Authors:** Pan Chen, Hongliang Yao, Weiwei Su, Yuying Zheng, Weiyang Fan, Liping Zhang, Tingting Chen, Shuling Wu, Weijian Zhang, Yan He, Zenghao Yan, Yonggang Wang, Peibo Li

**Affiliations:** ^1^ Guangdong Engineering and Technology Research Center for Quality and Efficacy Re-evaluation of Post-marketed TCM, Guangdong Key Laboratory of Plant Resources, School of Life Sciences, Sun Yat-sen University, Guangzhou, China; ^2^ Guangdong Key Laboratory of Animal Conservation and Resource Utilization, Guangdong Public Laboratory of Wild Animal Conservation and Utilization, Drug Synthesis and Evaluation Center, Guangdong Institute of Applied Biological Resources, Guangzhou, China

**Keywords:** oral ulcers, Kouyanqing Granule, sleep deprivation, neuro-immuno-endocrine system, oxidative stress, tryptophan metabolism

## Abstract

Oral ulcers are the most prevalent oral mucosal diseases globally, and no specific treatment schemes are currently available due to the complexity of oral ulcer diseases. Sleep deprivation increases the risk of a deterioration in oral health. Kouyanqing Granule (KYQG) has been used for decades in China to treat inflammatory diseases of the mouth and throat associated with the hyperactivity of fire due to yin deficiency syndrome. However, the mechanisms underlying the effects of KYQG in the treatment of oral ulcers are still unclear. The aims of this study were to investigate whether KYQG treatment could attenuate the symptoms of oral ulcers worsened by sleep deprivation and identify the involved metabolic pathways. First, we conducted chemical profiling of KYQG *via* UPLC–MS analysis. We then combined pharmacological and metabolomics approaches in a phenol-induced rat model of oral ulcers worsened by sleep deprivation. A total of 79 compounds were initially identified. Our observations showed that KYQG treatment induced a significantly higher healing rate in oral ulcers worsened by sleep deprivation. KYQG significantly reduced the levels of 5-HT and GABA in serum, and only decreased the 5-HT level in brain tissue after phenol injury followed by sleep deprivation. Moreover, KYQG administration significantly suppressed systemic inflammation by inhibiting TNF-α, IL-1β, IL-6, IL-18, and MCP-1. Immunohistochemical analysis further revealed that KYQG inhibited IL-6 expression in buccal mucosa tissues. KYQG treatment also significantly decreased the serum levels of ACTH, CORT, IgM, and 8-OHdG. Serum metabolomics analysis showed that a total of 30 metabolites showed significant differential abundances under KYQG intervention, while metabolic pathway analysis suggested that the altered metabolites were associated with the dysregulation of eight metabolic pathways. Taken together, our results indicated that KYQG attenuates the symptoms of oral ulcers worsened by sleep deprivation probably through the regulation of the neuroimmunoendocrine system, oxidative stress levels, and tryptophan metabolism. This study also provides a novel approach for addressing the increased health risks resulting from sleep deficiency using an herbal medicine formula.

## Introduction

Oral ulcers are among the most painful and common mucosal diseases. These conditions have uncertain and complex etiology, including mechanical injuries, immunologic dysregulation, genetic predisposition, hormonal level fluctuations, systemic diseases, microelement deficiencies, nutritional imbalances, microbial infections, allergic factors, adverse drug reactions, and psychological stress (Scully and Shotts, 2000; Guimaraes et al., 2007; Akintoye and Greenberg, 2014). The breakdown of immune regulation at mucosal sites plays a crucial role in the development of oral ulcers. Recurrent aphthous stomatitis (RAS) is one of the most common oral ulcer diseases, and it has a prevalence in the general population that ranges between 5% and 60% (Taylor et al., 2014). Oral ulcers usually expose nerve endings in the underlying lamina propria and can severely affect patients’ quality of life. Due to their complexity, no specific or ideal treatment approach is currently available for the treatment of these diseases.

Traditional Chinese medicine (TCM) formulas are used for oral ulcer therapy, acting through the synergistic effects of multiple constituents. Kouyanqing Granule (KYQG), a TCM formula, comprises parts of five species of herbs, namely, the flower bud of Lonicera macrantha (D.Don) Spreng. (syn. *Lonicera macranthoides* Hand.-Mazz., 1936) (FL), tuberous root of *Ophiopogon japonicus* (Thunb.) Ker Gawl., 1807 (TO), root of *Scrophularia ningpoensis* Hemsl. (RS), tuberous fibrous root of *Asparagus cochinchinensis* (Lour.) Merr. (TA), and root of *Glycyrrhiza uralensis* Fisch. ex DC. (RG). In China, KYQG has been used for decades to treat inflammatory diseases of the mouth and throat, such as RAS, traumatic ulcers, oral leukoplakia, and oral lichen planus. KYQG is also recorded in the Chinese Pharmacopoeia of 2015 as a treatment for oral diseases associated with the hyperactivity of fire due to Yin deficiency (HFYD) syndrome (China, 2015). The symptoms of HFYD, a type of TCM syndrome, have characteristics of sleeplessness, thirst, dry mouth, and dysphoria with a feverish sensation (Poon et al., 2012; Jiang et al., 2015). Sleep loss usually leads to HFYD (Yan et al., 2009; Poon et al., 2012). Inadequate sleep is a prevalent issue in today’s society and can increase the risk of numerous disorders, such as cardiovascular disease (Tobaldini et al., 2017), obesity (Fatima et al., 2015), diabetes (Lee et al., 2017), inflammatory bowel disease (Ananthakrishnan et al., 2013), pregnancy complications (Romero and Badr, 2014), hypertension (Pepin et al., 2014), and neurobehavioral and cognitive impairment (Kreutzmann et al., 2015). Epidemiological evidence indicates that a link exists between sleep loss and oral health, such as the increased risk of periodontitis (Lee et al., 2014) and gingivitis (Carra et al., 2017) that results from sleep deprivation. We have previously shown that sleep deprivation exacerbates the symptoms of oral ulcers and delays the healing process in the rat, and that oxidative stress and the neuro-immuno-endocrine system may have roles in worsening oral ulcer symptoms (Chen et al., 2019). However, the mechanisms underlying the therapeutic effects of KYQG against oral ulcers associated with the HFYD syndrome remain poorly understood. To address this, we performed chemical profiling of KYQG *via* UPLC–MS and investigated the treatment effects and potential mechanisms by combining pharmacological and metabolomics approaches in a phenol-induced rat model of oral ulcers worsened by sleep deprivation.

## Materials and Methods

### Chemical Profiling Based on UPLC–MS Analysis

The reference standards of lysine, arginine, aspartic acid, and citrulline were purchased from Jianglai Biotechnology co., LTD (Shanghai, China). Chelidonic acid and diosmetin were purchased from Sigma‐Aldrich (St Louis, MO, USA). Phenylalanine, chlorogenic acid, caffeic acid, liquiritin, isoquercitrin, angoroside C, isoliquiritoside, harpagoside, macranthoidin B, luteolin, quercetin, and formononetin were purchased from the National Institute for the Control of Pharmaceutical and Biological Products (Beijing, China). KYQG extracts (Batch number: A19M025) were provided by Hutchison Whampoa Guangzhou Baiyunshan Chinese Medicine Co., Ltd (Guangzhou, China), and KYQG was prepared by extracting the five herbs in the following proportion: flower bud of Lonicera macrantha (D.Don) Spreng. (syn. *Lonicera macranthoides* Hand.-Mazz., 1936) (FL), 26%; tuberous root of *Ophiopogon japonicus* (Thunb.) Ker Gawl., 1807 (TO), 21%; root of *Scrophularia ningpoensis* Hemsl. (RS), 21%; tuberous fibrous root of *Asparagus cochinchinensis* (Lour.) Merr. (TA), 21%; root of *Glycyrrhiza uralensis* Fisch. ex DC. (RG), 11%, as described in our previous study (Liu et al., 2015). One gram of KYQG extract was ultrasonically extracted with 80% methanol for 30 min. The mixture was precipitated overnight and filtered (pore size 0.22 μm; Jinteng, China) before chemical profiling analyses. The UPLC system was equipped with a Shimadzu CBM-20Alite controller. Chromatographic separation of the sample was conducted on a Dionex Bonded Silica C18 column (4.6 mm × 150 mm, 3 μm; Sunnyvale, California, USA) maintained at 40 °C. The mobile phase consisted of acetonitrile containing 0.1% formic acid (mobile phase A) and water containing 0.1% formic acid (mobile phase B) and the gradient elution program was as follows: 0–7 min, 2 to 10% A; 7–95 min, 10% to 41% A; 95–105 min, 41% to 100% A; 105–115 min, 100% A. The ﬂow rate was 0.3 mL/min, and the sample injection volume was 3 μL. The mass spectral analyses were performed using a hybrid triple quadrupole time-of-flight mass spectrometer (Triple TOF™ 5600+, AB Sciex, Forster City, CA, USA). The electrospray ionization (ESI) source was operated in both positive and negative ion mode with the following settings: ion spray voltage was 5500 V in positive ion mode and 4500 V in negative mode, ion source gas 1 = 55 psi, ion source gas 2 = 55 psi, curtain gas = 35 psi, and collision gas pressure = 10 psi. The components of the KYQG sample were identified *via* the mass spectrum data, chromatographic retention time (RT), MS/MS fragmentation pathway, and comparisons with available standards, references, and a mass spectral library (Natural Products HRMS/MS Spectral Library, Version 1.0; AB Sciex).

### Quantification Analysis

To perform a quantification analysis, 1 mg of KYQG extract was ultrasonically extracted with 9 ml of 50% methanol in volumetric flask (10 ml) for 30 min and then diluted to volume. The mixture was diluted to appropriate concentration filtered through a 0.22 µm filter for quantification analysis. The stock solutions of arginine, liquiritin, chlorogenic acid, harpagoside, luteolin-7-O-glucoside, and glycyrrhizic acid were prepared in methanol and then mixed and diluted to serial concentrations in 50% methanol. Linearity of the method was examined by using the serial mixed standards and the calibration curves were calculated by the least squares linear regression method. The separation of the sample was conducted on a Phenomenex Kinetex C18 100A (2.10mm × 100 mm, 2.6 μm, Torrance, USA). The mobile phase consisted of acetonitrile containing 0.1% formic acid (mobile phase A) and water containing 0.1% formic acid (mobile phase B), and the gradient elution program was as follows: 0–30 min, 5 to 62% A; 30–33 min, 62% to 100% A. The ﬂow rate was 0.3 mL/min, and the sample injection volume was 5 μL. MS conditions were set as described above (Part 2.1).

### Animals and Treatment

**Figure 1:**
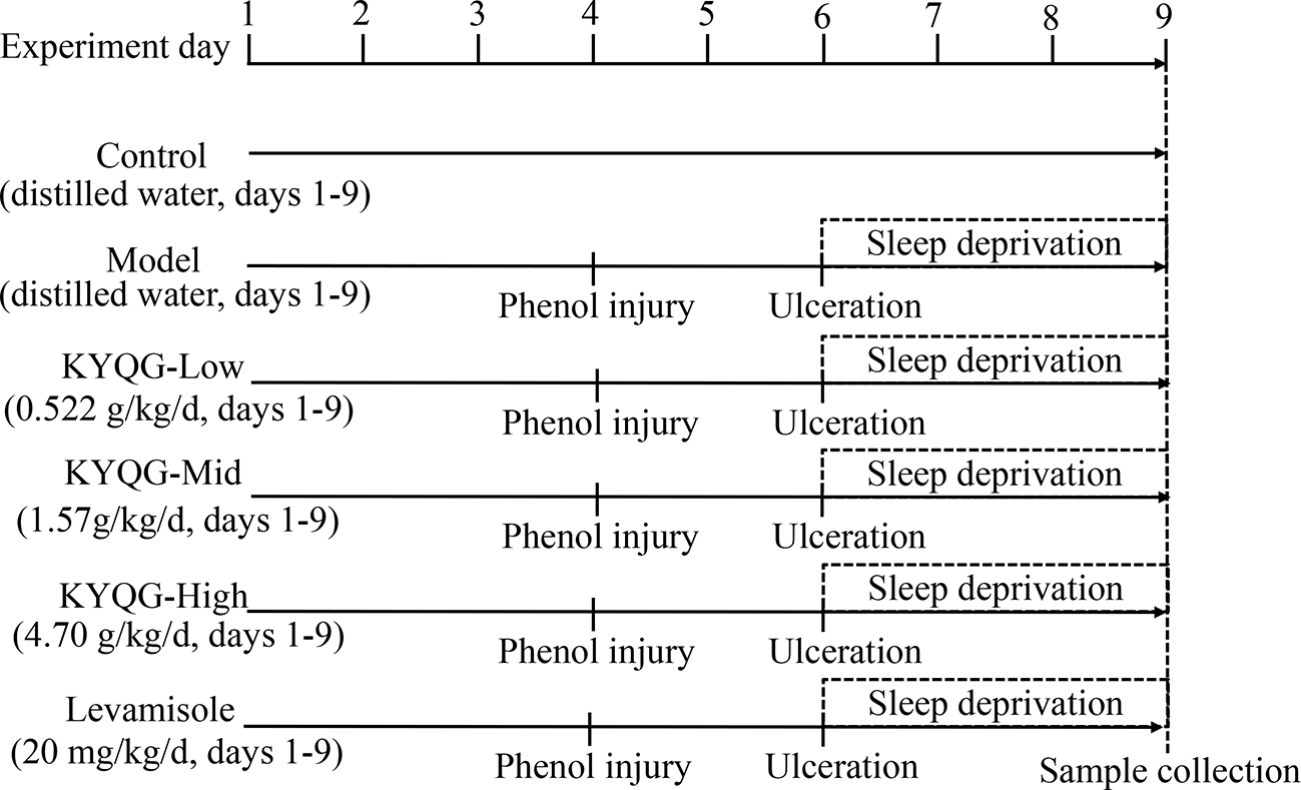
Schematic representation of the experimental protocol showing the sequence of events throughout the experiment.

Eight-week-old male Sprague–Dawley rats were purchased from the Laboratory Animal Center of Sun Yat-sen University. The animals were randomly divided into six groups (*n* = 7 per group): a Control group, Model group, KYQG-Low group, KYQG-Mid group, KYQG-High group, and Levamisole group. The KYQG-Low, KYQG-Mid, and KYQG-High groups were treated with KYQG extract at the doses of 0.522, 1.57, and 4.70 g/kg/d, respectively, by oral administration from day 1 to day 9. The Levamisole group was treated with levamisole (20 mg/kg/d, levamisole solution was prepared at a concentration of 2 mg/ml in water and then intragastrically administered to rats at a dose of 10 ml/kg/d), and the other groups were treated with distilled water. On day 4, oral ulcers were induced on the cheek mucosa of rats in the Model group, KYQG groups, and Levamisole group by phenol-induced chemical injury, as described in our previous study (Chen et al., 2019). Ulcers that were almost uniformly round formed in the oral mucosal region on day 6. The rats in the Model group, KYQG groups, and Levamisole group were then subjected to sleep deprivation for 72 h using a modified multiple platform technique as described in our previous study (Chen et al., 2019). At the end of the sleep deprivation period, all the animals were anesthetized, and blood was collected from the abdominal aorta. The blood was centrifuged at 5,000 rpm for 20 min at 4°C to obtain serum, which was stored at −80°C. Whole brains and buccal mucosa tissues were collected and immediately frozen at −80°C. The animal experimental protocol is depicted in **Figure 1**. All animal experiments were approved by the Institutional Animal Care and Use Committee of Sun Yat-sen University (Approval No. SYSU-IACUC-2019-000181). All efforts were made to minimize the suffering of the experimental animals.

### Measurement of Ulcer Area

Measurement of the ulcer area was started on day 6 after induction of ulceration using Image-Pro Plus 6.0 (Media Cybernetics Inc., Rockville, MD, USA) as described in our previous study (Chen et al., 2019). The degree of healing was expressed as ulcer healing rate = (A_6_ − A_t_)/A_6_ × 100%, where A_6_ and A_t_ represent the initial ulcer area and the ulcer area at the time of observation, respectively.

### Enzyme-Linked Immunosorbent Assay (ELISA)

The serum concentrations of tumor necrosis factor-alpha (TNF-α), interleukin (IL)-1β, IL-6, monocyte chemoattractant protein 1 (MCP-1), adrenocorticotropin hormone (ACTH), corticosterone (CORT), immunoglobulin M (IgM), and 8-hydroxy-deoxyguanosine (8-OHdG) were measured using commercial ELISA kits (Nanjing Jiancheng Bioengineering Institute, Nanjing, Jiangsu, China) according to the manufacturer’s instructions.

### Measurement of Neurotransmitter Levels

The levels of *γ*-aminobutyric acid (GABA) and 5-hydroxytryptamine (5-HT) in serum and whole-brain tissues were determined using ultra-high performance liquid chromatography–tandem mass spectrometry as described in our previous study (Chen et al., 2019).

### Immunohistochemistry

Immunohistochemistry was performed to determine the expression of IL-6 in healthy buccal mucosa tissues. All specimens were fixed in 4% paraformaldehyde (BioSharp, Hefei, China), embedded in paraffin (Guangdong Dachuan special wax Co. Ltd, Maoming, China), and sliced into 5-μm sections. Subsequently, the sections were dewaxed with xylene and dehydrated with ethanol. The processed sections were preincubated with 3% H_2_O_2_ in PBS for 10 min to block endogenous peroxidases. The sections were rinsed three times with PBS (5 min each wash) and then blocked with 5% (*w/v*) bovine serum albumin for 30 min at 37°C. Then, the sections were incubated with an anti-IL-6 primary antibody (1:5000, ab9324, Abcam, Cambridge, UK) at 37°C for 2 h, followed by incubation with the secondary antibody at 37°C for 30 min. Subsequently, the samples were treated with diaminobenzidine tetrahydrochloride. To stop the reaction and clear unbound antibody, the incubated sections were rinsed three times with PBS before and after the corresponding treatments. Staining was developed by incubation in a diaminobenzidine solution for 10 min followed by staining with hematoxylin for 5 min. Images of these sections were obtained using an optical microscope (Olympus BX43; Olympus Co., Tokyo, Japan). The mean optical density of each field (epithelial tissue section) was represented as integrated optical density (IOD)/area using Image-Pro Plus 6.0.

### Metabolomics Analysis of Serum Samples

Metabolomic profiling was performed using LC–QTOF/MS. For the untargeted metabolomics analysis, an LC system (Shimadzu CBM20Alite controller) combined with a hybrid triple quadrupole time-of-flight mass spectrometer (Triple TOF™ 5600+, AB Sciex) was used. A Kinetex^®^ C18 100A column (2.6 μm, 150 mm × 3.0 mm, Phenomenex Corp, Torrance, CA, USA) was used for sample separation. Water containing 0.1% formic acid (*v/v*) (mobile phase A) and acetonitrile (mobile phase B) were used at a flow rate of 0.3 mL/min. The gradient elution program was as follows: 0–20 min, 5% to 80% B; 20–30 min, 80% to 90% B; 30–40 min, and 90% to 95% B. The serum samples of the Control, Model, and KYQG-High groups were used for metabolomics analysis. Each serum sample (100 µL) was added to 200 µL of a cold acetonitrile/methanol solution (1:1, *v/v*) to precipitate the proteins. After vortexing for 5 min, the solution was centrifuged at 13,000 rpm for 20 min at 4°C. Afterwards, 10 µL of the supernatant was injected into the LC–QTOF/MS system for analysis. The mass spectrometry data were acquired in negative ion mode, and the electrospray source settings were as follows: gas 1 = 55 psi, gas 2 = 55 psi, source temperature = 550°C, and ion spray voltage was −4500 V with 35 psi curtain gas. Samples were analyzed with scanning mass-to-charge (*m/z*) ratio ranging from 50 to 1500 Da (30 V collision energy, 15 eV collision energy spread, and 80 V declustering potential). Nitrogen was used as nebulizer and auxiliary gas.

The raw data were converted to the mzXML format using ProteoWizard and processed using the R package ‘XCMS’ for peak detection, extraction, alignment, and integration. Then, an in-house MS2 database was applied in metabolite annotation. The cutoff for annotation was set at 0.3. The metabolomics data were analyzed by orthogonal partial least squares discriminant analysis (OPLS-DA) using SIMCA (version 15.0.2, Umea, Sweden). Furthermore, the value of variable importance in the projection (VIP) of the first principal component in OPLS-DA analysis was obtained and summarizes the contribution of each variable to the model. Metabolites with VIP >1 and *P* < 0.05 (Student’s *t*-test) were considered as having significantly changed abundance. In addition, the KEGG (http://www.genome.jp/kegg/) and MetaboAnalyst (http://www.metaboanalyst.ca/) databases were used for pathway enrichment analysis.

### Statistical Analysis

Data analyses were performed using GraphPad Prism 8.0.2 (GraphPad Prism Software, La Jolla, CA, USA). The results are presented as means ± SD for each group. To assess the differences between the groups, one-way ANOVA was carried out followed by Tukey test. Results were considered significant at *P* < 0.05.

## Results

### Chemical Profiling and Quantification Analysis of KYQG

The retention time, exact molecular mass, and MS/MS data for each compound were obtained and applied in the KYQG chemical profiling analysis performed using Peakview software. The total ion chromatograms of KYQG in the positive and negative modes are shown in **Supplementary Figure S1**. A total of 79 compounds were initially identified, including phenolics, flavonoids, triterpenoid saponins, and amino acids. The identified compounds are shown in **Table 1**, and the mass spectrum data of standards are shown in **Supplementary Figure S2** and **Supplementary Table S1**. The results of quantification analysis of six components in KYQG are shown in **Tables 2** and **3**. The linear correlations of the calibration curves over the test range were acceptable. Chlorogenic acid was the most abundant component among the six components detected.

**Table 1:** Identification of the chemical composition of Kouyanqing Granule (KYQG).

No.	RT (min)	Formula	[M+H]^+^(Error, ppm)	[M–H]^–^(Error, ppm)	MS/MS fragments (Positive mode)	MS/MS fragments (Negative mode)	Identification	Plant material
1	5.28	C_6_H_14_N_2_O_2_	147.1128 (–0.4)		130.0867 [M+H–NH_3_]^+^, 84.0826 [M+H–NH_3_–HCOOH]^+^, 67.0566 [M+H–2NH_3_–HCOOH]^+^		Lysine	TA/TO
2	5.63	C_6_H_14_N_4_O_2_	175.1190 (0.0)	173.1058 (+8.1)	158.0934 [M+H–NH_3_]^+^, 130.0985 [M+H–NH_3_–CO]^+^, 116.0716 [M+H–CN_3_H_5_]^+^ 70.0681 [M+H–CN_3_H_5_–HCOOH]^+^, 60.0594	156.0767 [M–H–NH_3_]^–^, 131.0827 [M–H–C_3_H_6_]^–^	Arginine	TA/TO
3	5.74	C_4_H_8_N_2_O_3_	133.0606 (+0.3)		116.0336 [M+H–NH_3_]^+^, 87.0565 [M+H–HCOOH]^+^, 74.0259 [M+H–C_2_H_5_NO]^+^, 70.0312 [M+H–HCOOH–NH_3_]^+^		Asparagine	TA/TO
4	5.84	C_4_H_7_NO_4_		132.0315 (+9.7)		115.0045 [M–H–NH_3_]^–^, 88.0413 [M–H–CO_2_]^–^, 71.0154 [M–H– CO_2_–NH_3_]^–^	Aspartic acid	TA/TO
5	5.89	C_5_H_13_NO	104.1074 (+3.5)		60.0836 [M+H-C_2_H_4_O]^+^, 58.0681 [M+H–H_2_O–C_2_H_4_]^+^		Choline	TA/TO
6	6.14	C_6_H_13_N_3_O_3_	176.1029 (–0.5)		159.0765 [M+H–NH_3_]^+^, 113.0709 [M+H–NH_3_–HCOOH]^+^, 70.0673 [M+H–NH_3_–CO_2_–CH_3_NO]^+^		Citrulline	TA/TO
7	6.21	C_4_H_9_NO_2_	104.0709 (–0.1)		87.0457 [M+H–NH_3_]^+^, 69.0359 [M+H–NH_3_–H_2_O]^+^, 60.0835 [M+H–CO_2_]^+^		γ-Aminobutyric acid	TA/TO
8	6.46	C_30_H_52_O_26_		827.2671 (–0.3)		545.1759 [M–H–C_10_H_18_O_9_]^–^, 383.1197 [M–H–C_10_H_18_O_9_–Glc]^–^, 179.0539 [M–H–4Glc]^–^	MDG–1	TO
9	6.51	C_7_H_7_NO_2_	138.0550 (+0.7)		94.0662 [M+H–CO_2_]^+^, 92.0506 [M+H–HCOOH]^+^, 78.0355 [M+H–CO_2_–CH_4_]^+^, 65.0412 [M+H–CO_2_–NCH_3_]^+^		Trigonelline	FL/RG
10	6.59	C_5_H_9_NO_2_	116.0709 (+0.8)		70.0677 [M+H–HCOOH]^+^, 68.0521		Proline	TA/TO
11	6.64	C_24_H_42_O_21_		665.2142 (+0.9)		485.1530 [M–H–Glc–H_2_O]^–^, 383.1197 [M–H–Glc–C_4_H_8_O_4_]^–^, 341.1085 [M–H–2Glc]^–^, 179.0558 [M–H–3Glc]^–^,	Stachyose	TA/TO/RG
12	6.75	C_10_H_17_N_3_O_6_	276.1188 (–0.8)		147.0763 [M+H–C_5_H_7_NO_3_]^+^, 130.0497 [M+H–C_5_H_10_N_2_O_3_]^+^		QGE dipeptide	TA/TO
13	6.82	C_12_H_22_O_11_		341.1088 (+0.7)		179.0560 [M–H–Glc]^–^, 161.0459 [M–H–Glc–H_2_O]^–^, 119.0346,89.0249	Sucrose/maltose	TA/TO
14	6.85	C_30_H_52_O_26_		827.2671 (–0.3)		545.1759[M–H–Glc–C_4_H_8_O_4_]^–^, 383.1197 [M–H–2Glc–C_4_H_8_O_4_]^–^	Verbascose	TA/RS
15	6.91	C_18_H_32_O_16_		503.1610 (+1.1)		383.1205 [M–H–C_4_H_8_O_4_]^–^, 341.1092 [M–H–Glc]^–^, 221.0650 [M–H–Glc–C_4_H_8_O_4_]^–^, 179.0558 [M–H–2Glc]^–^, 89.0250 [M–H–2Glc–C_3_H_6_O_3_]^–^	Raffinose	TA/TO
16	7.13	C_5_H_5_N_5_	136.0619 (+0.6)		119.0358 [M+H–NH_3_]^+^, 92.0252 [M+H–NH_3_–HCN]^+^, 65.0161 [M+H–CH_2_N_2_–CH_3_N]^+^		Adenine	FL/RG/RS
17	10.21	C_6_H_5_NO_2_	124.0396 (+0.6)		106.0289 [M+H–H_2_O]^+^, 80.0514 [M+H–CO_2_]^+^, 78.0357 [M+H–HCOOH]^+^,		Nicotinic acid	FL
18	10.61	C_7_H_4_O_6_	185.0082 (+0.7)	182.9939 (+2.1)	141.0173 [M+H–CO_2_]^+^, 97.0297 [M+H–2CO_2_]^+^, 71.0152 [M+H–2CO_2_–C_2_H_2_]^+^	139.0038 [M–H–CO_2_]^–^, 68.9988 [M–H–C_4_H_2_O_4_]^–^, 67.0207 [M–H–2CO_2_–CO]^–^	Chelidonic acid	TA
19	10.86	C_5_H_11_O_2_NS	150.0582 (–1.0)		133.0298 [M+H–NH_3_]^+^, 104.0534 [M+H–HCOOH]^+^, 102.0549 [M+H–CH_4_S]^+^, 61.0129 [M+H–C_3_H_9_NO_2_]^+^, 56.0529 [M+H–CH_4_S–HCOOH]^+^		Methionine	TA/TO
20	11.61	C_5_H_7_NO_3_	130.0500 (+1.1)		84.0461 [M+H–HCOOH]^+^, 56.0531 [M+H–HCOOH–CO]^+^		Pyroglutamic acid	TA/FL/RG
21	12.16	C_4_H_4_N_2_O_2_	113.0348 (+1.8)		96.0090 [M+H–NH_3_]^+^ 70.0309 [M+H-CHNO]^+^		Uracil	FL/RG
22	12.81	C_10_H_13_N_5_O_4_	268.1043 (+1.0)		136.0620 [M+H–Rib]^+^, 119.0357 [M+H– Rib–NH_3_]^+^		Adenosine	RS
23	12.95	C_9_H_11_NO_3_	182.0811 (–0.2)		165.0543 [M+H–NH_3_]^+^, 147.0435 [M+H–NH_3_–H_2_O]^+^, 136.0755 [M+H–HCOOH]^+^, 119.0490 [M+H–HCOOH–NH_3_]^+^, 91.0553 [M+H–HCOOH–NH_3_–CO]^+^, 77.0403,65.04111		Tyrosine	TA/TO
24	13.37	C_10_H_13_N_5_O_5_	284.0992 (+0.9)		152.0569 [M+H–Rib]^+^, 135.0298 [M+H– Rib–NH_3_]^+^		Guanosine	RS
25	16.27	C_15_H_24_O_10_		363.1291 (+0.9)		201.0759 [M–H–Glc]^–^, 183.0656 [M–H–Glc–H_2_O]^–^, 165.0549 [M–H–Glc–2H_2_O]^–^, 139.0388 [M–H–Glc–2H_2_O–C_2_H_2_]^–^,	Harpagide	RS
26	17.03	C_9_H_11_NO_2_	166.0864 (+0.9)	164.0725 (+4.6)	120.0816 [M+H–HCOOH]^+^, 103.0554 [M+H–HCOOH–NH_3_]^+^	147.0445 [M–H–NH_3_]^–^, 103.0555 [M–H–NH_3_–CO_2_]^–^, 72.0096	Phenylalanine	TA/TO/RS
27	19.16	C_8_H_8_O_4_		167.0361 (+1.2)		123.0445 [M–H–CO_2_]^–^, 93.0351 [M–H–CO_2_–OCH_2_]^–^	Vanillic acid	TO/RG
28	21.83	C_16_H_18_O_9_	355.1025 (+0.3)	353.0877 (–0.2)	163.0396 [M+H–C_7_H_12_O_6_]^+^, 145.0288 [M+H–C_7_H_12_O_6_–H2O]^+^, 117.0342 [M+H–C_7_H_12_O_6_–H_2_O–CO]^+^, 89.0402 [M+H–C_7_H_12_O_6_–H_2_O–2CO]^+^	191.0557 [M–H–C_9_H_6_O_3_]^–^, 179.0344 [M–H–C_7_H_10_O_5_]^–^, 135.0450 [M–H–C_7_H_10_O_5_–CO_2_]^–^	Neochlorogenic acid	FL
29	22.40	C_7_H_6_O_4_	155.0339 (0)	153.0195 (+0.9)	137.0242 [M+H–H_2_O]^+^, 109.0279 [M+H–H_2_O–CO]^+^, 93.0352 [M+H–H_2_O–CO_2_]^+^, 81.0324 [M+H–H_2_O–2CO]^+^, 65.0413 [M+H–H_2_O–CO_2_–CO]^+^	109.0280 [M–H–CO_2_]^–^, 91.0178 [M–H–CO_2_–H_2_O]^–^	Protocatechuic acid	FL
30	22.76	C_21_H_28_O_13_	511.1404 (–3.5)	487.1444 (–2.8)	365.0824 [M+Na–Rha]^+^	179.0338 [M–H–Rha–Caffeoyl]^–^, 161.0224 [M–H–Caffeoyl–Rha–H_2_O]^–^ 135.0440 [Caffeic acid–CO_2_]^–^	Cistanoside F	RS
31	24.81	C_7_H_8_O_3_	141.0547 (0.2)		126.0309 [M+H–CH_3_]^+^, 97.0304 [M+H–CH_4_–CO]^+^, 81.0682 [M+H–CH_4_O–CO]^+^, 71.0144, 55.0230		5-Methoxymethyl furfural	TA
32	28.13	C_16_H_18_O_9_	355.1026 (+0.6)	353.0870 (–1.3)	163.0394 [M+H–C_7_H_12_O_6_]^+^, 145.0290 [M+H–C_7_H_12_O_6_–H_2_O]^+^, 117.0344 [M+H–C_7_H_12_O_6_–H_2_O–CO]^+^, 89.0405 [M+H–C_7_H_12_O_6_–H_2_O–2CO]^+^	191.0549 [M–H–C_9_H_6_O_3_]^–^	Chlorogenic acid	FL
33	29.64	C_16_H_18_O_9_	355.1027 (+0.9)	353.0869 (–0.5)	163.0390 [M+H–C_7_H_12_O_6_]^+^, 145.0284 [M+H–C_7_H_12_O_6_–H_2_O]^+^, 117.0338 [M+H–C_7_H_12_O_6_–H_2_O–CO]^+^	191.0548 [M–H–C_9_H_6_O_3_]^–^, 173.0442 [M–H–C_9_H_6_O_3_–H_2_O]^–^, 135.0441 [M–H–C_7_H_10_O_5_–CO_2_]^–^, 93.0345	Cryptochlorogenic acid	FL
34	33.42	C_8_H_8_O_3_	153.0546 (–0.2)	151.0409 (3.1)	135.1159 [M+H–H_2_O]^+^, 107.0866 [M+H–H_2_O–CO]^+^, 77.0399 [M+H–2CH_2_O]^+^,	136.0169 [M–H–CH_3_]^–^, 108.0210 [M–H–CH_3_–CO]^–^	Vanillin	RG/TO
35	34.92	C_9_H_8_O_4_	181.0409 (–0.7)	179.0354 (+2.5)	163.0387 [M+H–H_2_O]^+^, 135.0439 [M+H–HCOOH]^+^, 89.398 [M+H–H_2_O–CO–HCOOH]^+^	135.0446 [M–H–CO_2_]^–^	Caffeic acid	FL
36	36.14	C_16_H_18_O_8_		337.0926 (–0.9)		191.0556 [M–H–C_9_H_6_O_2_]^–^, 173.0437 [M–H–C_9_H_6_O_2_–H_2_O]^–^, 93.0347 [M–H–C_10_H_12_O_7_]^–^	5-(p-Coumaroyl) quinic acid	FL
37	39.11	C_9_H_8_O_3_	165.0545 (–0.9)		121.0286 [M+H–CO_2_]^+^, 77.0405 [M+H–H_2_O–C_3_H_2_O_2_]^+^,		p-coumaric acid	FL
38	39.15	C_17_H_20_O_9_		367.1014 (–3.8)		191.0552 [M–H–C_7_H_12_O_5_]^–^, 173.0446 [M–H–C_7_H_12_O_5_–H_2_O]^–^, 93.0340 [M–H–C_11_H_14_O_7_–H_2_O]^–^	3-O-Caffeoylquinic acid methyl ester	FL
39	43.08	C_27_H_32_O_14_	581.1847 (–1.5)	579.1703 (–1.0)	419.1332 [M+H–Glc]^+^, 257.0802 [M+H–2Glc]^+^, 239.0745 [M+H–2Glc–H_2_O]^+^	417.1207 [M–H–Glc]^–^, 297.0661 [M–H–C_14_H_18_O_6_]^–^, 255.0647 [M–H–2Glc]^–^, 135.0066 [M–H–2Glc–RDA]^–^	Liquiritigenin 7,4’-diglucoside	RG
40	45.99	C_27_H_30_O_16_	611.1601 (–1.4)	609.1447 (–1.9)	465.1076 [M+H–Rha]^+^, 303.0502 [M+H–Glc–Rha]^+^, 287.0603,229.0507,85.0248	300.0258 [M–H–Glc–Rha]^–^, 271.0244 [M–H–Glc–Rha–CH_2_O]^–^	Rutin	FL
41	47.09	C_26_H_30_O_13_	551.1742 (–1.0)	549.1610 (–0.7)	419.1273 [M+H–Api]^+^, 257.0794 [M+H–Api–Glc]^+^, 145.0480, 137.0230 [M–H–C_19_H_26_O_10_]^–^	255.0659 [M–H–Api–Glc]^–^, 135.0084 [M–H–C_19_H_26_O_10_]^–^	Liquiritin apioside	RG
42	48.23	C_27_H_30_O_15_	595.1643 (–0.7)	593.1491 (–3.4)	287.0541 [M+H–Rut]^+^	285.0388 [M–H–Rut]^–^	Kaempferol-3-O-rutinoside	FL
43	48.33	C_29_H_36_O_15_		623.1976 (–0.9)		461.1678 [M–H–O–Rha]^–^, 161.0235 [M–H– Rha–C_8_H_9_O_2_–C_9_H_7_O_4_]^–^, 133.0289 [M–H– Rha–C_8_H_9_O_2_–C_9_H_7_O_4_–CO]^–^	Acteoside	RS
44	48.41	C_21_H_22_O_9_	419.1334 (–0.7)	417.1189 (–0.6)	257.0803 [M+H–Glc]^+^, 137.0224 [M+H–C_14_H_18_O_6_]^+^	255.0645 [M–H–Glc]^–^, 135.0073 [M–H–Glc–RAD]^–^, 119.0489	Liquiritin	RG
45	49.18	C_21_H_20_O_12_	465.1027 (–0.1)	463.0876 (–1.2)	303.0489 [M+H–Glc]^+^	301.0351 [M–H–Glc]^–^, 271.0240 [M–Glc–CH_2_O]^–^, 255.0285 [M–H–Glc–O–CH_2_O]^–^, 151.0016 [M–H–Glc–RDA]^–^	Isoquercitrin	RG
46	49.25	C_21_H_20_O_11_	449.1077 (–0.3)	447.0917 (–2.3)	287.0554 [M+H–Glc]^+^	285.0404 [M–H–Glc]^–^	Luteolin-7-O-glucoside	FL/RG
47	52.22	C_29_H_36_O_15_		623.1975 (–1.0)		461.1675 [M–H–O–Rha]^–^, 161.0238 [M–H–C_20_H_28_O_11_–H_2_O]^–^, 135.0440 [M–H–C_20_H_28_O_11_–H_2_O–C_2_H_2_]^–^	Isoacteoside	RS
48	53.10	C_25_H_24_O_12_	517.1335 (–1.0)	515.1188 (–1.3)	499.1231 [M+H–H_2_O]^+^, 163.0389 [M+H–C_6_H_18_O_9_]^+^ 145.0286 [M+H–C_6_H_18_O_9_–H_2_O]^+^	353.0861 [M–H–C_9_H_6_O_3_]^–^, 191.0546 [M–H–2C_9_H_6_O_3_]^–^, 173.0439 [M–H–2C_9_H_6_O_3_–H_2_O]^–^,	3,4-Dicaffeoylquinic acid	FL
49	55.23	C_21_H_20_O_11_	449.1077 (–0.3)	447.0919 (–3.2)	287.0552 [M+H–Glc]^+^	285.0387[M–H–Glc]^–^, 255.0284 [M–H–Glc–CH_2_O]^–^, 227.0327 [M–H–Glc–CH_2_O–CO]^–^	Quercitrin	FL
50	55.74	C_25_H_24_O_12_	517.1325 (–0.6)	515.1177 (–2.1)	499.1229 [M+H–H_2_O]^+^, 163.0390 [M+H–C_6_H_18_O_9_]^+^, 145.0280 [M+H–C_6_H_18_O_9_–H_2_O]^+^	353.0867 [M–H–C_9_H_6_O_3_]^–^, 191.0547 [M–H–2C_9_H_6_O_3_]^–^, 173.0336 [M–H–2C_9_H_6_O_3_–H_2_O]^–^,	3,5-Dicaffeoylquinic acid	FL
51	56.82	C_36_H_48_O_19_	807.2675 (–0.9)	783.2723 (+0.7)	807.2675 [M+Na]^+^	607.2276 [M–H–C_6_H_11_O_4_–CHO]^–^ 193.0492 [M–H–C_26_H_38_O_15_]^–^, 175.0383 [M–H–C_26_H_38_O_15_–H_2_O]^–^	Angoroside C	RS
52	56.98	C_21_H_20_O_10_	433.1127 (–0.5)	431.0965 (–4.4)	271.0614 [M+H–Glc]^+^	269.0446 [M–H–Glc]^–^,	Afzelin	RG/FL
53	58.88	C_25_H_24_O_12_	517.1336 (–0.8)	515.1181 (–0.9)	499.1240 [M+H–H_2_O]^+^, 163.0397 [M+H–C_16_H_18_O_9_]^+^, 145.0289 [M+H–C_16_H_18_O_9_–H_2_O]^+^	353.0853 [M–H–C_9_H_6_O_3_]^–^, 191.0541 [M–H–2C_9_H_6_O_3_]^–^, 173.0435 [M–H–2C_9_H_6_O_3_–H_2_O]^–^,	4,5-Dicaffeoylquinic acid	FL
54	62.84	C_26_H_30_O_13_	551.1762 (+0.5)	549.1595 (–3.3)	419.1367 [M+H–Api]^+^, 257.0813 [M+H–Api–Glc]^+^, 239.0680 [M+H– Api–Glc–H_2_O]^+^,	255.0649 [M–H–Api–Glc]^–^, 135.0074 [M–H–Api–Glc–C_6_H_4_–CO]^–^	Isoliquiritin apioside	RG
55	66.06	C_22_H_22_O_9_	431.1335 (–0.4)	475.1224 (–1.3)	269.0809 [M+H–Glc]^+^	267.0639 [M–H–Glc]^–^, 252.0403 [M–H–Glc–CH_3_]^–^	Ononin	RG
56	66.21	C_21_H_22_O_9_	419.1334 (–0.7)	417.1179 (–2.9)	257.0812 [M+H–Glc]^+^, 137.0224 [M+H–Glc–C_8_H_6_O]^+^	255.0648 [M–H–Glc]^–^, 148.0150,135.0077, 119.0489 [M–H–Glc–C_8_H_6_O–H_2_O]^–^, 92.0260 [M–H–Glc–C_9_H_7_O_3_]^–^	Isoliquiritoside	RG
57	73.66	C_24_H_30_O_11_	517.1675 (–1.0)	493.1679 (–1.8)	369.1170,203.0533	345.1161 [M–H–C_9_H_7_O_2_]^–^, 147.0434 [M–H–C_15_H_22_O_9_]^–^, 165.0535,103.0544	Harpagoside	RS
58	73.68	C_9_H_8_O_2_	149.0598 (–0.6)		121.0650 [M+H–CO] ^–^, 93.0713 [M+H–2CO] ^–^		Dihydrocoumarin	RG
59	73.75	C_25_H_32_O_13_		539.1745 (+1.0)		493.1679 [M–H–HCOOH]^–^, 345.1169 [M–H–C_10_H_10_O_4_]^–^, 147.0429	8-O-Feruloylharpagide	RS
60	74.42	C_15_H_12_O_4_	257.0810 (–2.3)	255.0658 (–3.9)	239.0675 [M+H–H_2_O]^+^, 211.0752 [M+H–H_2_O–CO]^+^, 147.0440 [M+H–C_6_H_6_O_2_]^+^, 137.0232 [M+H–C_8_H_8_O]^+^	135.0081 [M–H–C_8_H_8_O]^–^, 119.0500 [M–H–C_7_H_4_O_3_]^–^	Liquiritigenin	RG
61	75.02	C_65_H_106_O_32_		1397.6516 (–1.1)		1073.5549 [M–H–2Glc]^–^, 744.3308	Macranthoidin B	FL
62	76.60	C_15_H_10_O_6_	287.0552 (–0.5)	285.0393 (–2.1)	153.0169 [M+H–C_8_H_6_O_2_]^+^	175.0389 [M–H–C_6_H_6_O_2_]^–^, 133.0289 [M–H–C_7_H_4_O_4_]^–^	Luteolin	TO/FL
63	77.19	C_47_H_76_O_17_	913.5130 (+0.8)		781.4760 [M-Ara]^+^, 751.4601 [M+H–Glc]^+^, 455.3512 [M+H– Ara–Rha–Glc]^+^, 437.3398 [M+H–Ara–Rha–Glc–H_2_O]^+^,		Macranthoside A	FL
64	77.23	C_59_H_96_O_27_		1235.5997 (–1.9)		911.5033 [M–H–2Glc]^–^	Macranthoidin A	FL
65	77.24	C_47_H_76_O_17_		911.4993 (–2.3)		749.4506 [M–H–Glc]^–^, 603.3903 [M–H–Glc–Rha]^–^	3-O-α-L-arabinopyranosyl(2→1)-O-α-L-rhamnopyranosyl-hederagenin-28-O-β-D -glucopyranosyl ester	FL
66	77.79	C_15_H_10_O_7_		301.0341 (–1.9)		178.9966 [M–H–C_7_H_6_O_2_]^–^, 151.0020 [M–H–C_8_H_6_O_3_]^–^	Quercetin	RG
67	79.70	C_53_H_86_O_22_		1073.5496 (–4.1)		749.4489 [M–H–2Glc]^–^, 323.0972[Mal]	Dipsacoside B	FL
68	81.62	C_47_H_76_O_18_		927.4934 (–2.0)		603.3911 [M–H–2Glc]^–^	Akebia saponin D	FL
69	89.34	C_17_H_14_O_7_	331.0811 (–0.3)	329.0648 (–3.3)	315.0507 [M+H–OH]^+^, 316.0569 [M+H–CH_3_]^+^, 203.0333 [M+H–C_6_H_8_O_3_]^+^, 153.0168 [M+H–C_10_H_10_O_3_]^+^	299.0171 [M–H–CHO]^–^, 271.0235 [M–H–CHO–CO]^–^, 243.0300 [M–H–CHO–2CO]^–^, 203.0353,161.0214	Tricin	FL
70	90.34	C_53_H_86_O_22_		1073.5437 (-6.4)		911.4934 [M–H–Glc]^–^	Macranthoside B	FL
71	90.99	C_16_H_12_O_6_	301.0706 (+0.7)	299.0544 (–3.7)	286.0473 [M+H–CH_3_]^+^, 258.0511 [M+H–CH_3_–CO]^+^, 119.0487,153.0189	284.0292 [M–H–CH_3_]^–^, 256.0335 [M–H–CH_3_–CO]^–^, 227.0349 [M–H–CH_3_–CO–CHO]^–^	Diosmetin	FL
72	95.18	C_42_H_68_O_14_		795.4455 (–3.3)		471.3444 [M–H–2Glc]^–^, 323.0961	Dipsacussaponin A	FL
73	96.33	C_16_H_12_O_6_	301.0707 (+0.8)	299.0536 (–0.9)	286.0467 [M+H–CH_3_]^+^, 285.0388 [M+H–OH]^+^, 258.0529 [M+H–CH_3_–CO]^+^, 121.0276 [M+H–C_7_H_8_O_2_–CO–CO]^+^	284.0294 [M–H–CH_3_]^–^, 255.0282 [M–H–CH_3_–CHO]^–^, 227.0333 [M–H–CH_3_–CHO–CO]^–^	Chrysoeriol	FL
74	100.92	C_42_H_62_O_16_	823.4098 (–1.1)		471.3453 [M+H–2C_6_H_8_O_6_]^+^, 453.3362 [M+H–2C_6_H_8_O_6_–H_2_O]^+^,	351.0554 [M–H–C_28_H_42_O_3_–CO_2_]^–^	Glycyrrhizic acid	RG
75	101.48	C_15_H_12_O_4_	257.0806 (0.7)	255.0646 (–0.8)	239.0696 [M+H–H_2_O]^+^, 137.0232 [M+H–C_8_H_8_O]^+^, 119.0500, 81.0353	135.0074 [M–H–C_8_H_8_O]^–^, 119.0492 [M–H– C_7_H_4_O_3_]^–^, 91.0183 [M–H–C_7_H_4_O_3_–CO]^–^,	Isoliquiritigenin	RG
76	102.69	C_16_H_12_O_4_	269.0809 (+0.3)	267.0645 (+0.7)	253.0491 [M+H–CH_3_]^+^, 197.0598 [M–CH_3_ –CO–CO]^+^, 181.0641 [M–OCH_3_ –CO–CO]^+^	252.0404 [M–H–CH_3_]^–^, 223.0373 [M–H–CH_3_–CHO]^–^, 195.0418 [M–H–CH_3_–CHO–CO]^–^, 132.0190 [M–H–C_7_H_7_O– CO]^–^, 91.0175 [M–H–C_9_H_8_O– CO–OH]^–^	Formononetin	RG
77	104.50	C_21_H_20_O_6_	369.1335 (+0.6)		313.0705 [M+H–2CO]^+^, 271.0607 [M+H–CO–CO–C2H2O]^+^, 211.0391, 215.0698 [M+H–C_6_H_6_O_2_–CO_2_]^+^		Glycycoumarin	RG
78	106.36	C_41_H_66_O_12_		749.4381 (–0.2)		603.3814 [M–H–Rha]^–^	Sapindoside A	FL
79	107.93	C_21_H_22_O_4_		337.1413 (–3.6)		305.1168 [M–H–CH_2_O]^–^	4′-O-methylglabridin	RG

The losses are Glc, glucose moiety; Rha, rhamnose moiety; Ara, arabinose moiety; Api, apiose moiety; Rib, ribose moiety; Mal, maltose.

**Table 2:** Regression equation of UHPLC-TOF-MS method for the quantification analysis.

Name	Regression equation	R²	Linear range(μg/ml)
Chlorogenic acid	y = 461220x + 170230	0.9985	0.44~43.76
Luteolin-7-O-glucoside	y= 1651116 x + 169046	0.9987	0.10~5.05
Arginine	y = 803438x + 245940	0.9955	0.11~11.05
Harpagoside	y = 715849x – 42021	0.9999	0.08~8.48
Liquiritin	y = 1392671 x + 590609	0.9920	0.11~11.45
Glycyrrhizic acid	y = 1459525 x + 456361	0.9912	0.20~10.09

**Table 3:** The quantification results of six components in KYQG.

Name	Peak area	Dilution ratio	Contents (mg/g)
Chlorogenic acid	1.18E+06	10000	2.82
Luteolin-7-O-glucoside	2.30E+06	30	0.06
Arginine	4.22E+05	30	0.01
Harpagoside	4.67E+06	30	0.31
Liquiritin	4.97E+06	100	0.43
Glycyrrhizic acid	6.69E+06	100	0.64

### Effects of KYQG on Oral Ulcer Area

**Figure 2:**
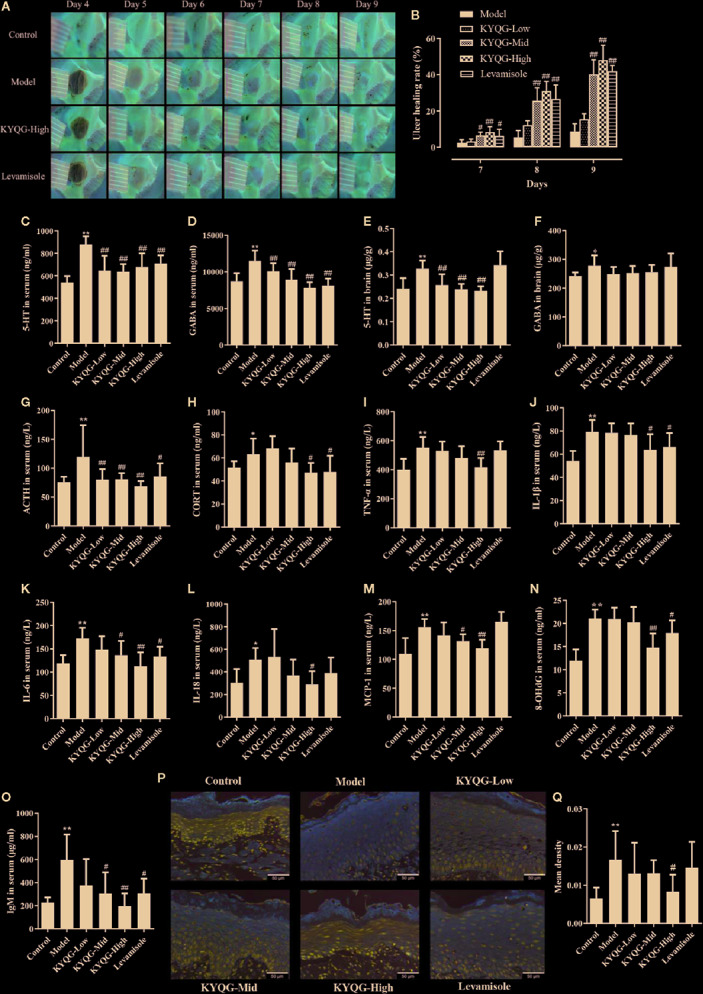
Results of the effect of Kouyanqing Granule (KYQG) in the treatment of phenol-induced oral ulcer worsened by sleep deprivation. Representative images of oral ulcers **(A)**; Effects of KYQG on the ulcer healing rate **(B)**, ulcer healing rate = [(A_6_ − A_t_)/A_6_] × 100%, where A_6_ represents the initial ulcer area on day 6, and A_t_ represents the ulcer area on days 7–9. Effects of KYQG on 5-hydroxytryptamine (5-HT) and *γ*-aminobutyric acid (GABA) levels in serum **(C, D)** and brain tissue **(E, F)**. Effects of KYQG on the serum levels of adrenocorticotropin hormone (ACTH) **(G)** and corticosterone (CORT) **(H)**. Effects of KYQG on the levels of inflammatory cytokines **(I–M)**, 8-hydroxy-2’-deoxyguanosine (8-OHdG) **(N)**, and immunoglobulin M (IgM) **(O)**. Interleukin (IL)-6 protein expression in epithelial tissue sections of buccal mucosa by immunohistochemistry in Control group, Model group, Kouyanqing Granule (KYQG)-Low group, KYQG-Mid group, KYQG-High group, and Levamisole group **(P)**. Quantification of IL-6 expression in epithelial tissue section of buccal mucosa **(Q)**. Data are expressed as means ± SD. ^*^ *P* < 0.05, ^**^ *P* < 0.01 compared with the Control group; ^#^ *P* < 0.05, ^##^ *P* < 0.01 compared with the Model group.

Representative images of buccal mucosa regions are shown in **Figure 2A**. On day 6, baseline ulcer areas of the Model, KYQF-Low, KYQF-Mid, KYQF-High, and Levamisole groups were 12.15 ± 0.68, 12.20 ± 0.66, 11.99 ± 0.74, 12.16 ± 1.05, and 12.18 ± 0.50 mm^2^, respectively, and the differences were not significant. The ulcer healing rate was calculated to assess the treatment effects of KYQG (**Figure 2B**). The oral ulcer area was decreased in all groups on days 7–9, with the highest ulcer healing rate being found in the KYQG-High group; the KYQG-Mid group, KYQG-high group, and Levamisole group showed significantly higher ulcer healing rates than the Model group on days 7–9. However, the difference between the Model group and the KYQG-Low group on days 7–9 was not significant.

### Effects of KYQG on Serum and Brain Neurotransmitter Levels

As shown in **Figures 2C–F**, KYQG treatment decreased the 5-HT level in serum and brain tissue and the GABA level in serum. However, compared with the Model group, KYQG treatment did not lead to a reduction in the GABA level in brain tissue.

### Effects of KYQG on the Serum Levels of ACTH and CORT

**Figures 2G, H** show the serum levels of ACTH and CORT. Levamisole and KYQG treatments resulted in a significant decrease in the serum level of ACTH. The serum level of CORT was also significantly reduced in the KYQG (4.70 g/kg/d)- and Levamisole-treated groups when compared with that in the Model group. The above results demonstrated that the protective effect of KYQG in oral ulcers may be modulated through the hypothalamic-pituitary-adrenocortical (HPA) axis.

### Effects of KYQG on the Levels of Inflammatory Cytokines, 8-OHdG, and IgM

As shown in **Figures 2I–O**, at the concentration of 4.70 g/kg/d, KYQG significantly reduced the serum levels of TNF-α, IL-1β, and IL-18, while at the concentrations of 1.57 and 4.70 g/kg/d, KYQG treatment lowered the serum levels of IL-6 and MCP-1. KYQG treatment also led to reduced serum levels of 8-OHdG and IgM, indicating that KYQG reduced oxidative stress and was involved in the regulation of immunological responses.

### Effects of KYQG on IL-6 Expression in Buccal Mucosa Tissues

To investigate the protective effects of KYQG on oral ulcers, the expression of IL-6 in epithelial tissue sections was investigated by immunohistochemistry. As illustrated in **Figures 2P, Q**, the expression of IL-6 in epithelial tissue sections from the Model group was significantly increased when compared with that of the Control group; however, IL-6 level was markedly lower in the KYQG-High group than in the Model group.

### Metabolic Profiling Analysis

**Figure 3:**
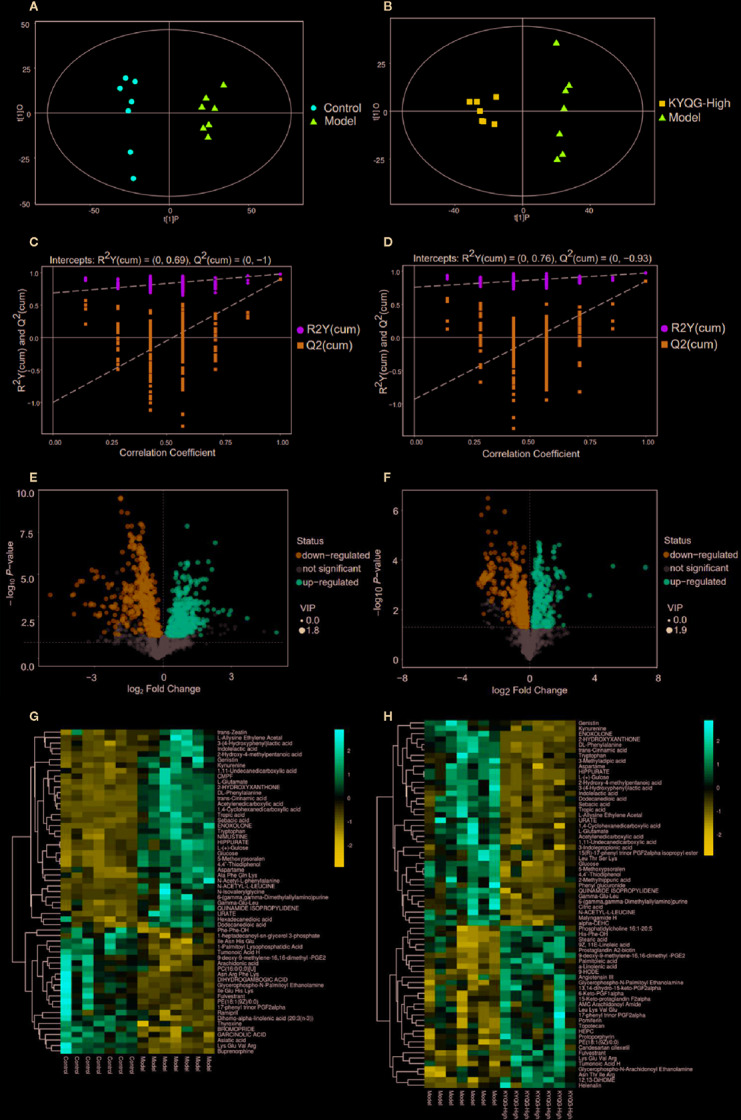
Multivariate statistical analysis of the serum metabolites. Orthogonal partial least squares discriminant analysis (OPLS-DA) score plot for the Control group *vs* the Model group **(A)** OPLS-DA score plot for the Model group *vs* the Kouyanqing Granule (KYQG) group **(B)**; Permutation test for the validity of the OPLS-DA model for the Control group *vs* the Model group **(C)** Permutation test for the validity of the OPLS-DA model for the Model group *vs* the KYQG group **(D)**. Volcano plots for the Control group *vs* the Model group **(E)** and Model group *vs* Kouyanqing Granule (KYQG)-High group **(F)** each point represents a metabolite, and the point size represents the variable importance in the projection (VIP) value of this metabolite in the orthogonal partial least squares discriminant analysis (OPLS-DA) model. Heat map of the hierarchical clustering analysis of the differential metabolites for the Control group *vs* the Model group **(G)** and the Model group *vs* the KYQG-High group **(H)** each column represents one serum sample, and each row represents one differential metabolite; the color represents the relative level of the differential metabolite with a gradient from blue (low levels) to red (high levels).

Serum metabolic profiling in the Control group, Model group, and KYQG-High group was assessed by multivariate analysis. A clear separation was observed among the Control, Model, and KYQG-High groups in OPLS-DA score plots (**Figures 3A, B**), suggesting differential metabolic profiles between two groups. A permutation test was used to verify the model to avoid the transition fit of the OPLS-DA mode (**Figures 3C, D**). The quantification parameters for this OPLS-DA model (R2Y and Q2) were all positive, indicating that the OPLS-DA model was robust.

### Identification of Metabolites and Pathway Analysis

**Figure 4:**
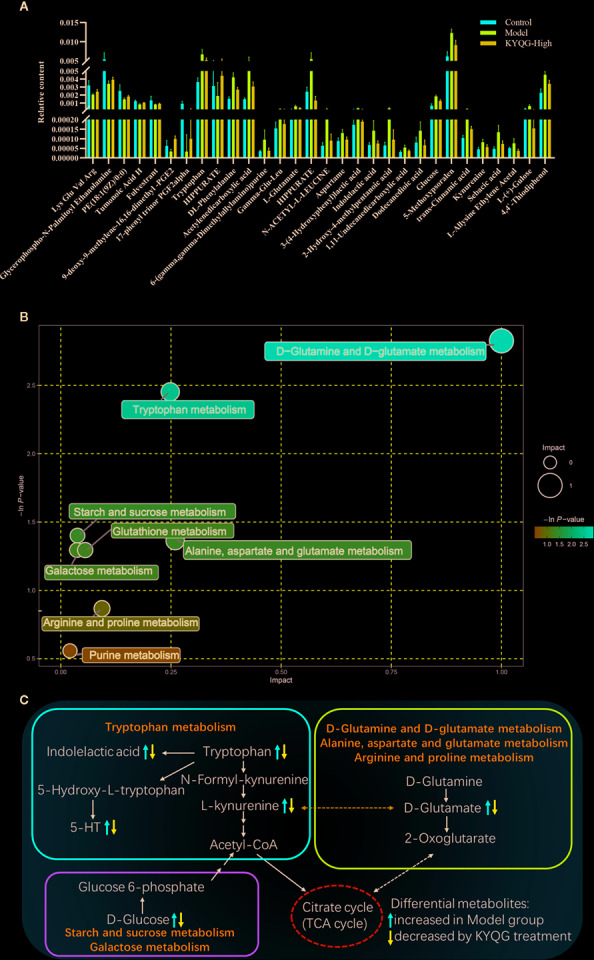
The relative levels of 30 regulated serum metabolites in rats after Kouyanqing Granule (KYQG) treatment **(A)**. Metabolic pathway analysis results based on the 30 regulated serum metabolites after Kouyanqing Granule (KYQG) treatment **(B)**. Summaries of metabolic pathways **(C)**.

According to the OPLS-DA analysis, the levels of the metabolites identified in the Model group were significantly different from those in the Control and KYQG-High groups. The metabolites showing significantly differential abundance were visualized through volcano plots (**Figures 3E, F**). A total of 59 differential metabolites were identified in the Model and Control groups, and 68 in the Model and KYQG groups, using a VIP >1.0 in the OPLS-DA models and a *p*-value <0.05 (two-tailed Student’s *t*-test). The generated heat map clearly depicts the relationships between these differential metabolites in different samples (**Figures 3G, H**). Based on the heatmaps, the Control and Model groups, as well as the Model and KYQG groups, could be completely separated. A total of 30 metabolites showed significantly differential abundance with KYQG treatment (**Figure 4A**), and these metabolites were used for pathway analysis. The metabolic pathway analysis using Metaboanalyst based on KEGG database (www.kegg.jp) revealed that eight pathological processes were associated with KYQG treatment, including D-Glutamine and D-glutamate metabolism, tryptophan metabolism, alanine, aspartate, and glutamate metabolism, arachidonic acid metabolism, and arginine and proline metabolism (**Figure 4B**). The important differential metabolites were summarized in **Figure 4C**, and we found that tryptophan metabolism plays an important role in the protection of KYQG against phenol-induced oral ulcer worsened by sleep deprivation.

## Discussion

In this study, we have combined data obtained by different experimental methods, and explored the associations between oral ulcer progression, metabolomics, inflammation, oxidative stress, neurotransmitter levels, and HPA activity in a rat model of oral ulcers.

We examined the effects of KYQG on the levels of inflammatory cytokines in our oral ulcer rat model. Our results demonstrated that KYQG could inhibit the increased levels of IL-1, IL-18, IL-6, and MCP-1 levels in serum. Notably, the expression of IL-6 in epithelial tissue sections was ameliorated by KYQG treatment. Excessive production of IL-6, IL-1β, and TNF-α is known to be associated with an increased risk of developing RAS (Bazrafshani et al., 2002; Guimaraes et al., 2007; Scully and Porter, 2008). Therefore, the inhibitory effects of KYQG on the production of inflammatory cytokines may account for its anti-oral ulcer activity.

Our data also showed that KYQG treatment inhibited the excessive release of ACTH and CORT when compared with the Model group. It has been shown that sleep deprivation can activate the HPA axis (Ganz, 2012; Opp and Krueger, 2015). However, the direct links between the HPA axis and oral ulcers are poorly understood, and further studies are required to elucidate whether the KYQG-mediated regulation of the HPA axis contributes to its anti-ulcer activity.

Our data suggested that KYQG could downregulate the levels of 5-HT and GABA in serum but could only decrease the 5-HT level in the brain. 5-HT, an important pronociceptive mediator, can induce inflammation and hyperalgesia or allodynia (Oliveira et al., 2007). Neurotransmitter levels are correlated with detrimental psychological factors, including depression and stress, and depression is one of the psychological factors with a role in the etiopathogenesis of oral ulcers (Alshahrani, 2014). This suggests that regulation of neurotransmitter levels by KYQG may contribute to its treatment effects. Furthermore, this result is consistent with the results of our serum metabolic analysis.

Metabolic analysis indicated that tryptophan, kynurenine, and indole lactic acid, metabolites involved in tryptophan metabolism, were highly induced in the Model group, and decreased after KYQG treatment. There are two main pathways for tryptophan metabolism, resulting in two different metabolites – 5-HT and kynurenine (Agus et al., 2018). The results of this preliminary study suggested that breakdown of tryptophan metabolism was involved in the pathogenesis of oral ulcers *via* both pathways. It is known that tryptophan metabolites, as aryl hydrocarbon receptor ligands, play important roles in the regulation of immune responses (Hubbard et al., 2015). In the present study, KYQG treatment also downregulated serum IgM levels. Neurotransmitters can also serve as immunomodulators (Pacheco et al., 2010). Numerous studies have confirmed the crucial role of immune responses in the pathogenesis of oral ulcers (Slebioda et al., 2014; Johnson et al., 2017). Tryptophan metabolism, oxidative stress, and inflammation have also been associated with various conditions, including neurological disorders and inflammatory bowel disease (Roager and Licht, 2018). Li et al. compared the salivary nontargeted metabolite profiles between healthy individuals and RAS patients and found that dysregulation of tryptophan metabolism was involved in the pathogenesis of this condition (Li Y. T. et al., 2018). A previous study reported that the tryptophan metabolism enzyme, L-kynureninase, is highly expressed in patients with chronic inflammatory skin disease (Harden et al., 2016). In summary, tryptophan metabolism plays a crucial role in the etiopathogenesis and therapeutics of oral ulcers, probably through the regulation of immune responses, inflammation, and oxidative stress; however, the exact mechanism through which tryptophan metabolism regulates the development of oral ulcers remains unknown and requires further study. According to the results of the metabolic pathway analysis, the level of D-glucose, a metabolite of energy metabolism, was higher in the Model group than in the Control group, but the levels were restored by KYQG treatment. Sleep deprivation has been reported to affect glucose metabolism (Knutson et al., 2007). However, the relationship between energy metabolism and oral ulcers is unclear, and whether KYQG-mediated regulation of energy metabolism contributes to its anti-oral ulcer effects requires further investigation. L-Glutamic acid is involved in four metabolic pathways, namely, those of D-glutamine and D-glutamate metabolism, alanine, aspartate and glutamate metabolism, glutathione metabolism, and arginine and proline metabolism. Increases in the levels of glutamic acid have been reported to reduce GSH levels and eventually stimulate ROS release in Riluzole-treated cells (Seol et al., 2016). In addition, the serum level of 8-OHdG was increased in rats of the Model group and decreased by KYQG treatment. Combined, these results indirectly suggest that KYQG reduces oxidative stress in the treatment of oral ulcers. A previous study reported that arginine and proline metabolism was the characteristic metabolic signature of dental plaque in patients with periodontal inflammation, which may be related to the metabolic profiles of disease-associated communities (Sakanaka et al., 2017). In addition, it has been suggested that an increase in the serum levels of uric acid represents an inflammatory response associated with obesity, metabolic syndrome, dysglycemic conditions, diabetes mellitus, hypertension, endothelial dysfunction, cardiovascular disease, and chronic kidney disease (Spiga et al., 2017). These results showed that comprehensive metabolomic profiling can provide further insights into the molecular mechanisms underlying the beneficial effects of KYQG on the symptoms of oral ulcers. Notably, the modulation of tryptophan metabolism by KYQG may be important for the treatment of oral ulcers. All the above changes suggest that KYQG may affect multiple aspects of the neuroimmunoendocrine system, which correlate with improved oral ulcer symptoms.

Our phytochemical analysis indicated that the main constituents of KYQG were phenolics, flavonoids, and triterpenoid saponins. The KYQG phenolics, such as neochlorogenic acid (Kim et al., 2015), chlorogenic acid (Naveed et al., 2018), cryptochlorogenic acid (Villalva et al., 2018), 3,4-dicaffeoylquinic acid (Wan et al., 2019), and 3,5-dicaffeoylquinic acid (Hong et al., 2015), reportedly exhibit antioxidant and anti-inflammatory activities. Isoquercitrin, a bioactive flavonoid in KYQG, possesses a variety of biological properties, including antioxidant (Li et al., 2016), anti-inflammatory (Rogerio et al., 2007), anti-ulcer, and hepatoprotective (Xie et al., 2016) effects. Luteolin-7-O-glucoside, another flavonoid constituent of KYQG, exhibits antioxidative activity through the regulation of the HO-1 signaling cascade in RAW 264.7 cells (Song and Park, 2014). Liquiritigenin has been reported to exert anti-inflammatory, antioxidant, antidiabetic, and antitumor activities (Lee et al., 2018). Liquiritin could protect against UVB−induced skin injury by inhibiting TLR4/MyD88/NF−κB mitogen−activated protein kinases and caspase pathways, which lead to alleviating the inflammatory response, oxidative stress, and apoptosis in mice and cells (Li X.-Q. et al., 2018).

Triterpenoid saponins such as glycyrrhizic acid, dipsacussaponin A, and akebia saponin D are also active constituents of KYQG. Glycyrrhizic acid reportedly inhibits inflammation by activating the glucocorticoid receptor and the PI3K/AKT/GSK3β signaling pathway (Kao et al., 2010), and it acts as a neuroprotectant in the postischemic brain by inhibiting HMGB1 phosphorylation and secretion (Kim et al., 2012). A different study reported that dipsacussaponin A inhibits TNF-α-induced NF-kB transcriptional activity in HepG2 cells (Quang et al., 2011). Akebia saponin D has been shown to inhibit amyloid beta-induced inflammatory responses and cognitive deficits in rats (Yu et al., 2012), and suppress corticosterone overproduction in an Alzheimer’s disease model (Wang et al., 2018). Amino acids such as lysine, tyrosine, and arginine potentially regulate immune function and promote wound healing (Li et al., 2007; Rahayu et al., 2016). Lysine has been reported to treat RAS (Singh et al., 2011), while arginine supplementation significantly improved the rate of healing of pressure ulcers (Desneves et al., 2005). Tyrosine could reportedly inhibit gelatin-induced inflammation (Meyers et al., 1979). It has shown that harpagoside and harpagide have an inhibitory effect on IFNγ/LPS-induced secretion of TNFα in differentiated THP-1 cells (Schopohl et al., 2016). We inferred that the diverse constituents of KYQG may be holistically responsible for the amelioration of oral ulcer symptoms worsened by sleep deprivation.

Metabolomics strategy was widely applied for investigation of pharmacodynamics (Zhang et al., 2019), screening of active constituents (Caesar et al., 2019), prediction of targets (Sun et al., 2019), discovery of quality-markers (Ding et al., 2018) in herbal medicines. In this study, we performed the chemical profiling of KYQG *in vitro* and investigated the treatment effects and biomarkers by combining pharmacological and metabolomics approaches. Nonetheless, an acknowledged limitation of this study is that the absorbed chemicals and their metabolites in serum have not been identified and quantified, which could be correlated with biomarkers to further elucidate the active constituents. Identification and quantification of the absorbed chemical markers of KYQG in serum are needed in future study.

In summary, our observations suggest that KYQG therapy could attenuate phenol-induced oral ulcers worsened by sleep deprivation in a rat model. Amelioration of symptoms was primarily associated with regulation of the neuroimmunoendocrine system, levels of oxidative stress, and tryptophan metabolism. The multiple functionalities of the diverse constituents of KYQG make it an efficient therapy for hard-to-treat oral ulcers. These findings may contribute to a better understanding of the clinical application of KYQG. This study also provides a novel approach for addressing the increased health risk resulting from sleep deficiency using an herbal medicine formula. Nevertheless, further studies are needed to elucidate the precise mechanisms by which KYQG ameliorates oral ulcer symptoms as well as the interactions between components and targets.

## Data Availability Statement

The datasets generated for this study are available on request to the corresponding author.

## Ethics Statement

The animal study was reviewed and approved by Institutional Animal Care and Use Committee, Sun Yat-sen University.

## Author Contributions

PL, HY, and WS provided the concept and designed the experiment. PC, YZ, WF, LZ, TC, WZ, ZY, YH, and SW performed the experiments. PC and YW analyzed the data. PC and PL wrote the manuscript. All authors contributed to the article and approved the submitted version.

## Funding

Financial support was provided by the Science and Technology Planning Project of Guangzhou, China (No. 201803010082) and Guangdong Academic of Sciences Special Project of Science and Technology Development (No. 2016GDASRC-0104).

## Conflict of Interest

The authors declare that the research was conducted in the absence of any commercial or financial relationships that could be construed as a potential conflict of interest.
