# Supplementary material for: Pharmacodynamic and Metabolomics Studies on the Effect of Kouyanqing Granule in the Treatment of Phenol-Induced Oral Ulcer Worsened by Sleep Deprivation
Source: Front Pharmacol. 2020 Jun 30;11:824. doi: 10.3389/fphar.2020.00824 (PMC7338550; doi:10.3389/fphar.2020.00824)
Supplement: Supplementary file 1 [file DataSheet_1.docx]

Supplementary Material

# Supplementary Figures and Tables

## Supplementary Figures


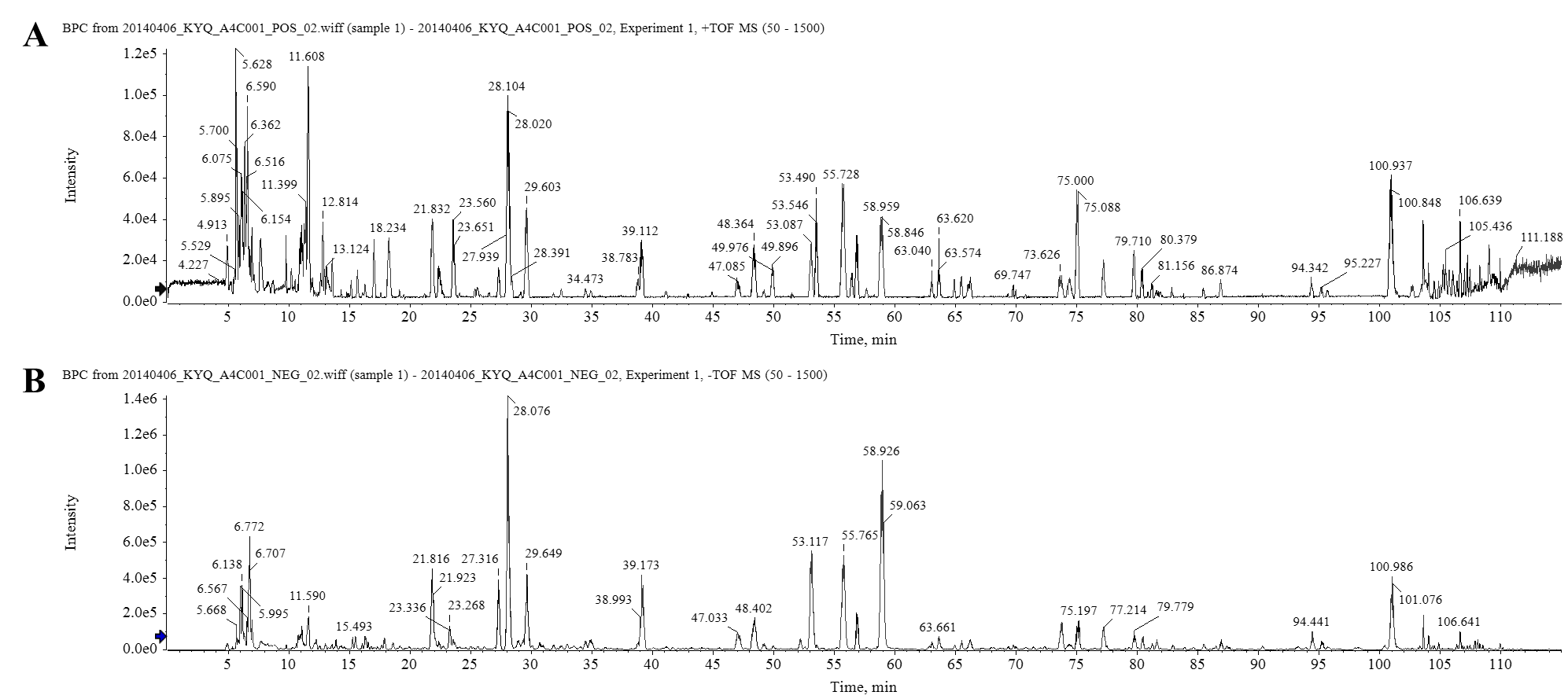


**Supplementary Figure 1.** Total ion chromatograms of Kouyanqing Granule (KYQG) in positive mode (A) and negative mode (B).

**
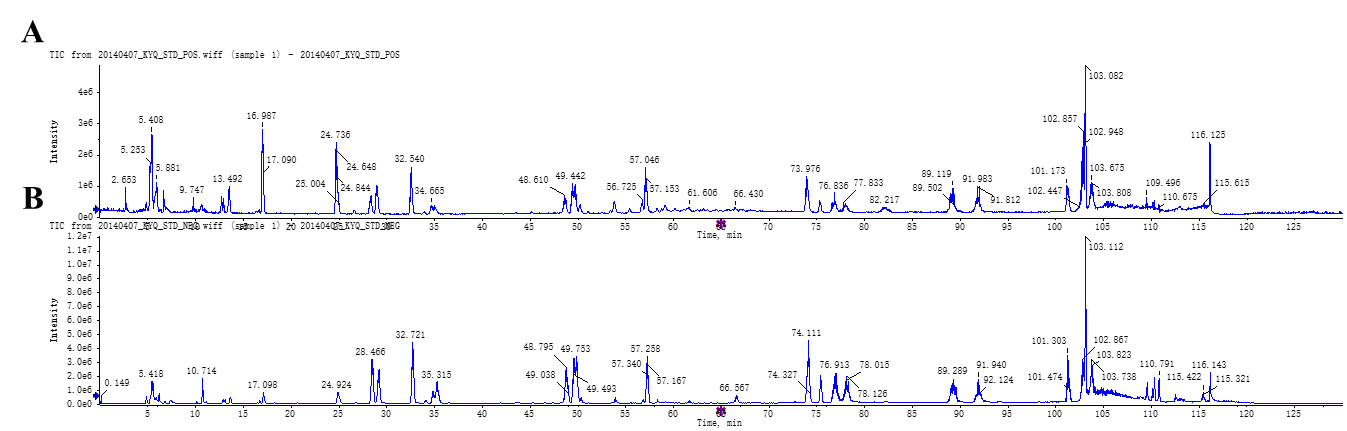
**

**Supplementary Figure 2.** Total ion chromatograms of standards in positive mode (A) and negative mode (B).

## Supplementary Tables

**Supplementary Table 1.** The mass spectrum data of standards. The losses are: Glc = glucose moiety.

| No. | RT  (min) | Formula | [M+H]+  (Error, ppm) | [M–H]–  (Error, ppm) | MS/MS fragments  (Positive mode) | MS/MS fragments  (Negative mode) | Standard |
| --- | --- | --- | --- | --- | --- | --- | --- |
| 1 | 5.25 | C_6_H_14_N_2_O_2_ | 147.1128  （+0.7） |  | 130.0868 [M+H–NH_3_]^+^,  84.0828 [M+H–NH_3_–HCOOH]^+^,  67.0573 [M+H–2NH_3_–HCOOH]^+^ |  | Lysine |
| 2 | 5.55 | C_6_H_14_N_4_O_2_ | 175.1188  （–0.9） | 173.1057  （+5.1） | 158.0921 [M+H–NH_3_]^+^,  130.0976 [M+H–NH_3_–CO]^+^,  116.0706 [M+H–CN_3_H_5_]^+^  70.0676 [M+H–CN_3_H_5_–HCOOH]^+^,  60.0586 | 156.0766 [M–H–NH_3_]^–^,  131.0828 [M–H–C_3_H_6_]^–^ | Arginine |
| 4 | 5.87 | C_4_H_7_NO_4_ |  | 132.0323  （+9.1） |  | 115.0045 [M–H–NH_3_]^–^,  88.0410 [M–H–CO_2_]^–^,  71.0151 [M–H– CO_2_–NH_3_]^–^ | Aspartic acid |
| 6 | 6.18 | C_6_H_13_N_3_O_3_ | 176.1031  （+0.8） |  | 159.0766 [M+H–NH_3_]^+^,  113.0718 [M+H–NH_3_–HCOOH]^+^,  70.0677 [M+H–NH_3_–CO_2_–CH_3_NO]^+^ |  | Citrulline |
| 18 | 10.71 | C_7_H_4_O_6_ | 185.0080  （–0.2） | 182.9946  （+6） | 141.0182 [M+H–CO_2_]^+^,  97.0294 [M+H–2CO_2_]^+^,  71.0151 [M+H–2CO_2_–C_2_H_2_]^+^ | 139.0043 [M–H–CO_2_]^–^,  68.9998 [M–H–C_4_H_2_O_4_]^–^,  67.0211 [M–H–2CO_2_–CO]^–^ | Chelidonic acid |
| 26 | 17.11 | C_9_H_11_NO_2_ | 166.0861  （–0.8） | 164.0730  （+7.7） | 120.0813 [M+H–HCOOH]^+^,  103.0552 [M+H–HCOOH–NH_3_]^+^ | 147.0454 [M–H–NH_3_]^–^,  103.0555 [M–H–NH_3_–CO_2_]^–^,  72.0111 | Phenylalanine |
| 32 | 28.48 | C_16_H_18_O_9_ | 355.1027  （+1） | 353.0877  （–0.4） | 163.0385 [M+H–C_7_H_12_O_6_]^+^,  145.0281 [M+H–C_7_H_12_O_6_–H_2_O]^+^,  117.0337 [M+H–C_7_H_12_O_6_–H_2_O–CO]^+^,  89.0392 [M+H–C_7_H_12_O_6_–H_2_O–2CO]^+^ | 191.0567 [M–H–C_9_H_6_O_3_]^–^ | Chlorogenic acid |
| 35 | 35.26 | C_9_H_8_O_4_ | 181.0409  （–0.7） | 179.0360  （+1.9） | 163.0387 [M+H–H_2_O]^+^,  135.0439 [M+H–HCOOH]^+^,  89.398 [M+H–H_2_O–CO–HCOOH]^+^ | 135.0455 [M–H–CO_2_]^–^ | Caffeic acid |
| 44 | 48.8 | C_21_H_22_O_9_ | 419.1334  （–0.7） | 417.1185  （–0.1.4） | 257.0808 [M+H–Glc]^+^,  137.0231 [M+H–C_14_H_18_O_6_]^+^ | 255.0665 [M–H–Glc]^–^,  135.0096 [M–H–Glc–RAD]^–^,  119.0512 | Liquiritin |
| 45 | 49.6 | C_21_H_20_O_12_ | 465.1022  （–0.6） | 463.0858  （–3.7） | 303.0490 [M+H–Glc]^+^ | 301.0343 [M–H–Glc]^–^,  271.0246 [M–Glc–CH_2_O]^–^,  255.0301 [M–H–Glc–O–CH_2_O]^–^,  151.0040 [M–H–Glc–RDA]^–^ | Isoquercitrin |
| 51 | 57.26 | C_36_H_48_O_19_ | 807.2675  （–0.9） | 783.2697  （–2.5） | 807.2675 [M+Na]^+^ | 607.2250 [M–H–C_6_H_11_O_4_–CHO]^–^  193.0506 [M–H–C_26_H_38_O_15_]^–^,  175.0396 [M–H–C_26_H_38_O_15_–H_2_O]^–^ | Angoroside C |
| 56 | 48.8 | C_21_H_22_O_9_ | 419.1334  （–0.7） | 417.1185  （–1.4） | 257.0808 [M+H–Glc]^+^,  137.0231 [M+H–Glc–C_8_H_6_O]^+^ | 255.0665 [M–H–Glc]^–^,  135.0096,  119.0512 [M–H–Glc–C_8_H_6_O–H_2_O]^–^,  92.0260 [M–H–Glc–C_9_H_7_O_3_]^–^ | Isoliquiritoside |
| 57 | 74.11 | C_24_H_30_O_11_ | 517.1675  （–1.0） | 493.1685  （–6.1） | 369.1170,203.0533 | 345.1167 [M–H–C_9_H_7_O_2_]^–^,  147.0451 [M–H–C_15_H_22_O_9_]^–^,  165.0553,103.0558 | Harpagoside |
| 61 | 75.42 | C_65_H_106_O_32_ |  | 1397.6516  （–1.1） |  | 1073.5549 [M–H–2Glc]^–^,  744.3308 | Macranthoidin B |
| 62 | 76.91 | C_15_H_10_O_6_ | 287.0553  （+0.8） | 285.0404  （–0.1） | 153.0181 [M+H–C_8_H_6_O_2_]^+^ | 175.00403 [M–H–C_6_H_6_O_2_]^–^,  133.0305 [M–H–C_7_H_4_O_4_]^–^ | Luteolin |
| 66 | 78.14 | C_15_H_10_O_7_ |  | 301.0305  （–0.7） |  | 178.9993 [M–H–C_7_H_6_O_2_]^–^,  151.0044 [M–H–C_8_H_6_O_3_]^–^ | Quercetin |
| 71 | 91.91 | C_16_H_12_O_6_ | 301.0708  （+0.4） | 299.0563  （–0.6） | 286.0477 [M+H–CH_3_]^+^,  258.0530 [M+H–CH_3_–CO]^+^,  119.0487,153.0189 | 284.0325 [M–H–CH_3_]^–^,  256.0388 [M–H–CH_3_–CO]^–^,  227.0363 [M–H–CH_3_–CO–CHO]^–^ | Diosmetin |
| 76 | 102.9 | C_16_H_12_O_4_ | 269.0810  （+0.5） | 267.0660  （–1.2） | 253.0551 [M+H–CH_3_]^+^,  197.0600 [M–CH_3_ –CO–CO]^+^,  181.0649 [M–OCH_3_ –CO–CO]^+^ | 252.0427 [M–H–CH_3_]^–^,  223.0407 [M–H–CH_3_–CHO]^–^,  195.0457 [M–H–CH_3_–CHO–CO]^–^,  132.0232 [M–H–C_7_H_7_O– CO]–,  91.0210 [M–H–C_9_H_8_O– CO–OH]^–^ | Formononetin |
